# Supplementary material for: Trust, Interaction with Neighbors, and Vaccination during the COVID-19 Pandemic: A Cross-Sectional Analysis of Chinese Data
Source: Vaccines (Basel). 2023 Aug 6;11(8):1332. doi: 10.3390/vaccines11081332 (PMC10459166; doi:10.3390/vaccines11081332)
Supplement: Supplementary file 1 [file vaccines-11-01332-s001.zip › vaccines-2528262-supplementary.pdf]

Table S1. Estimated results of multilevel fixed-effects models[a] (N = 6,860)

	General trust		Trust in the government		Interaction with neighbors	
	Coef.	95%CI	Coef.	95%CI	Coef.	95%CI
Female	0.020	(–0.004, 0.044)	0.007	(–0.008, 0.022)	–0.050	(–0.073, –0.028)
Age (ref. = 18–29 years)						
30–39 years	–0.011	(–0.062, 0.039)	0.026	(–0.006, 0.057)	0.026	(–0.022, 0.073)
40–49 years	0.036	(–0.018, 0.090)	0.018	(–0.016, 0.052)	0.030	(–0.021, 0.081)
50–59 years	0.093	(0.039, 0.147)	0.005	(–0.029, 0.039)	0.037	(–0.015, 0.088)
60–69 years	0.169	(0.111, 0.227)	0.030	(–0.006, 0.066)	0.057	(0.003, 0.112)
70–79 years	0.214	(0.149, 0.278)	0.005	(–0.036, 0.046)	0.080	(0.019, 0.141)
80 years +	0.276	(0.177, 0.375)	–0.040	(–0.102, 0.022)	0.085	(–0.008, 0.178)
Marital status (ref. = Married)						
Unmarried	0.019	(–0.030, 0.068)	0.001	(–0.030, 0.032)	–0.076	(–0.123, –0.029)
Divorced/separated	–0.010	(–0.049, 0.029)	–0.015	(–0.039, 0.009)	–0.017	(–0.054, 0.020)
Living alone	–0.055	(–0.093, –0.017)	–0.023	(–0.047, 0.001)	0.033	(–0.004, 0.069)
Educational attainment (ref. = Illiterate)						
Primary school	0.001	(–0.044, 0.046)	–0.009	(–0.037, 0.019)	–0.026	(–0.069, 0.016)
Junior high school	0.033	(–0.013, 0.079)	0.031	(0.002, 0.060)	–0.028	(–0.072, 0.015)
High school	0.061	(0.009, 0.112)	0.041	(0.009, 0.073)	–0.016	(–0.065, 0.032)
College or above	0.082	(0.023, 0.140)	0.028	(–0.009, 0.065)	–0.071	(–0.126, –0.015)
Occupation type (ref. = No work)						

Farming	0.020	(–0.018,	0.058)	–0.018	(–0.042,	0.006)	0.012	(–0.024,	0.048)
Government-related work[b]	0.051	(0.008,	0.094)	0.001	(–0.026,	0.028)	–0.049	(–0.090,	–0.009)
Private or foreign company	0.046	(0.006,	0.086)	–0.008	(–0.033,	0.017)	–0.067	(–0.105,	–0.030)
Self-employed	0.008	(–0.031,	0.047)	–0.007	(–0.032,	0.017)	–0.080	(–0.117,	–0.043)
Other	0.070	(–0.012,	0.152)	–0.025	(–0.077,	0.026)	–0.030	(–0.108,	0.048)
Family income (ref. = Lowest tertile)									
Second tertile	0.030	(–0.003,	0.063)	0.020	(0.000,	0.041)	0.015	(–0.016,	0.046)
Highest tertile	0.051	(0.013,	0.089)	0.025	(0.002,	0.049)	0.029	(–0.007,	0.065)
Unanswered	–0.040	(–0.076,	–0.004)	–0.024	(–0.046,	–0.001)	0.006	(–0.028,	0.040)
Poor self-rated health	–0.068	(–0.102,	–0.035)	–0.032	(–0.053,	–0.011)	–0.038	(–0.070,	–0.006)
Agricultural *hukou*	0.002	(–0.030,	0.034)	0.000	(–0.020,	0.021)	–0.019	(–0.050,	0.011)
Communist party member	0.075	(0.042,	0.108)	0.001	(–0.020,	0.022)	–0.016	(–0.048,	0.015)

[a] Further controlled for individual-, province- and community-level fixed effects.

[b] Encompassed works in 1) party and government institutions, 2) state-owned or collectively-owned enterprises, business groups, social groups, neighborhood or village committee, and 3) the army.

Table S2. Estimated results of multilevel logistic models to explain the probability of vaccination

	Young (N = 4,859)		Old (N = 2,001)	
	OR	95%CI	OR	95%CI
General trust				
Individual-level	1.35	(1.07, 1.70)	1.21	(0.90, 1.64)
Community-level	1.58	(1.14, 2.18)	1.17	(0.78, 1.75)
Female	0.54	(0.42, 0.68)	0.91	(0.68, 1.21)
Age (ref. = < 30 years / < 70 years)				
30-39 years	1.19	(0.81, 1.76)		-
40-49 years	2.08	(1.33, 3.24)		-
50-59 years	1.74	(1.12, 2.70)		-
60-69 years		-		-
70-79 years		-	0.51	(0.38, 0.69)
80 years +		-	0.18	(0.11, 0.30)
Marital status (ref. = Married)				
Unmarried	1.52	(1.01, 2.29)	0.43	(0.15, 1.27)
Divorced/separated	0.69	(0.45, 1.04)	0.81	(0.57, 1.16)
Living alone	0.69	(0.48, 1.00)	0.67	(0.45, 0.99)
Educational attainment (ref. = Illiterate)				
Primary school	1.27	(0.70, 2.29)	0.97	(0.66, 1.43)
Junior high school	1.47	(0.83, 2.60)	1.05	(0.68, 1.62)
High school	1.05	(0.58, 1.91)	1.19	(0.72, 1.97)

College or above	1.27	(0.67,	2.42)	1.13	(0.51,	2.50)
Occupation type (ref. = No work)						
Farming	1.18	(0.76,	1.83)	0.91	(0.62,	1.35)
Government-related work[a]	3.62	(2.27,	5.79)	0.66	(0.19,	2.32)
Private or foreign company	1.64	(1.18,	2.28)	1.50	(0.51,	4.36)
Self-employed	1.31	(0.94,	1.83)	0.94	(0.44,	2.02)
Other	1.23	(0.60,	2.53)	0.28	(0.07,	1.10)
Family income (ref. = Lowest tertile)						
Middle tertile	0.74	(0.51,	1.06)	0.78	(0.53,	1.15)
Highest tertile	0.97	(0.65,	1.44)	0.67	(0.42,	1.06)
Unanswered	0.67	(0.45,	0.99)	0.94	(0.64,	1.38)
Poor self-rated health	0.85	(0.57,	1.25)	0.53	(0.39,	0.73)
Agricultural *hukou*	1.20	(0.91,	1.57)	1.34	(0.91,	1.97)
Communist party member	1.77	(1.23,	2.54)	1.56	(1.04,	2.35)
Infection per capita at the province level (ref. = Lowest)						
Middle	1.00	(0.66,	1.51)	1.03	(0.58,	1.83)
High	0.87	(0.40,	1.87)	0.75	(0.26,	2.15)
Vaccination rate at the province level) (ref. = Lowest tertile)						
Middle tertile	4.35	(2.72,	6.96)	2.26	(1.16,	4.41)
Highest tertile	7.55	(4.79,	11.9)	7.66	(3.99,	14.7)

[a] Encompassed works in 1) party and government institutions, 2) state-owned or collectively-owned enterprises, business groups, social groups, neighborhood or village committee, and 3) the army.
